# Supplementary figures and images for: Validation of a simplified risk prediction model using a cloud based critical care registry in a lower-middle income country
Source: PLoS One. 2020 Dec 31;15(12):e0244989. doi: 10.1371/journal.pone.0244989 (PMC7775074; doi:10.1371/journal.pone.0244989)

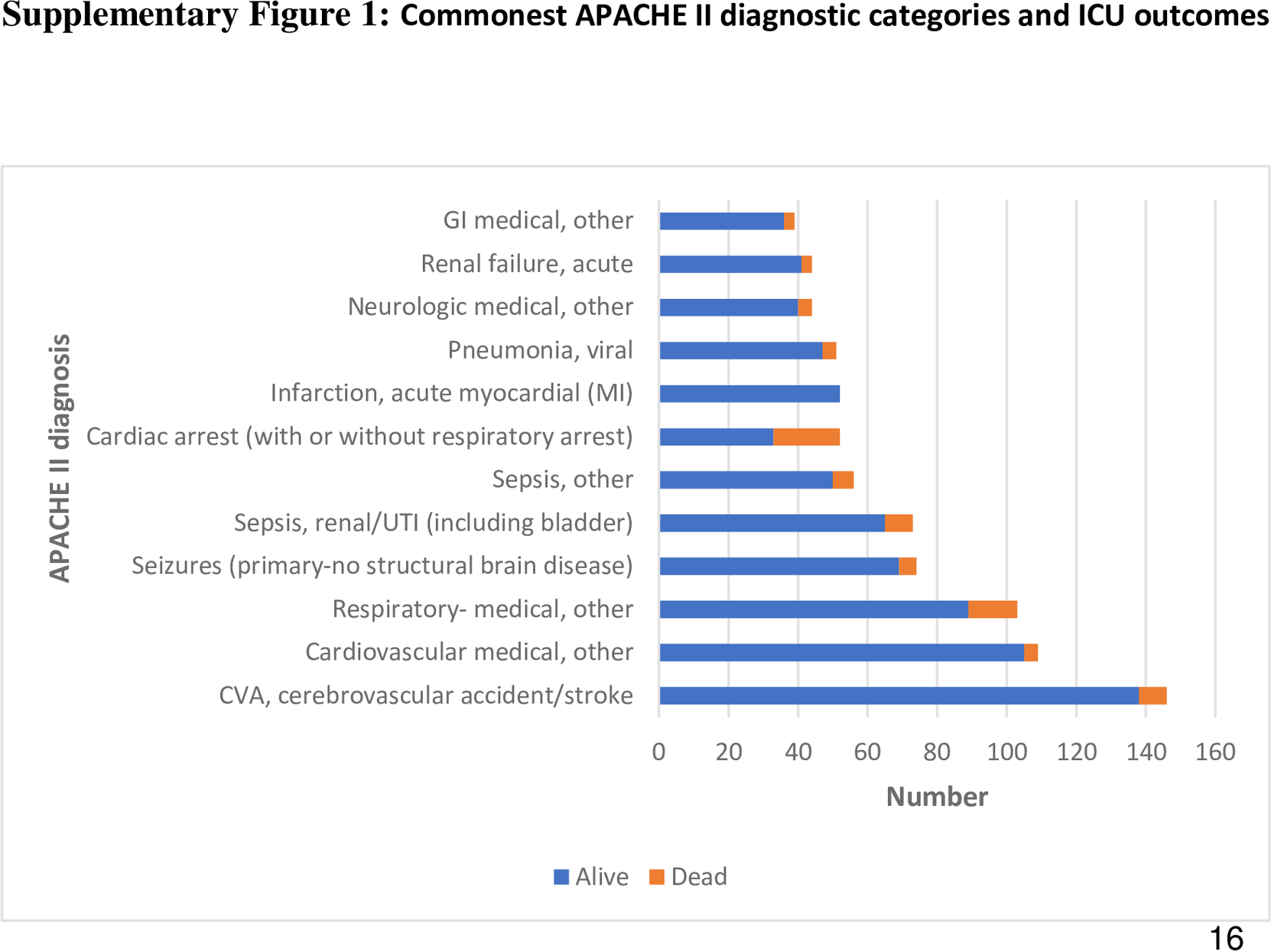

Supplement: S1 Fig — (TIF) [file pone.0244989.s001.tif]

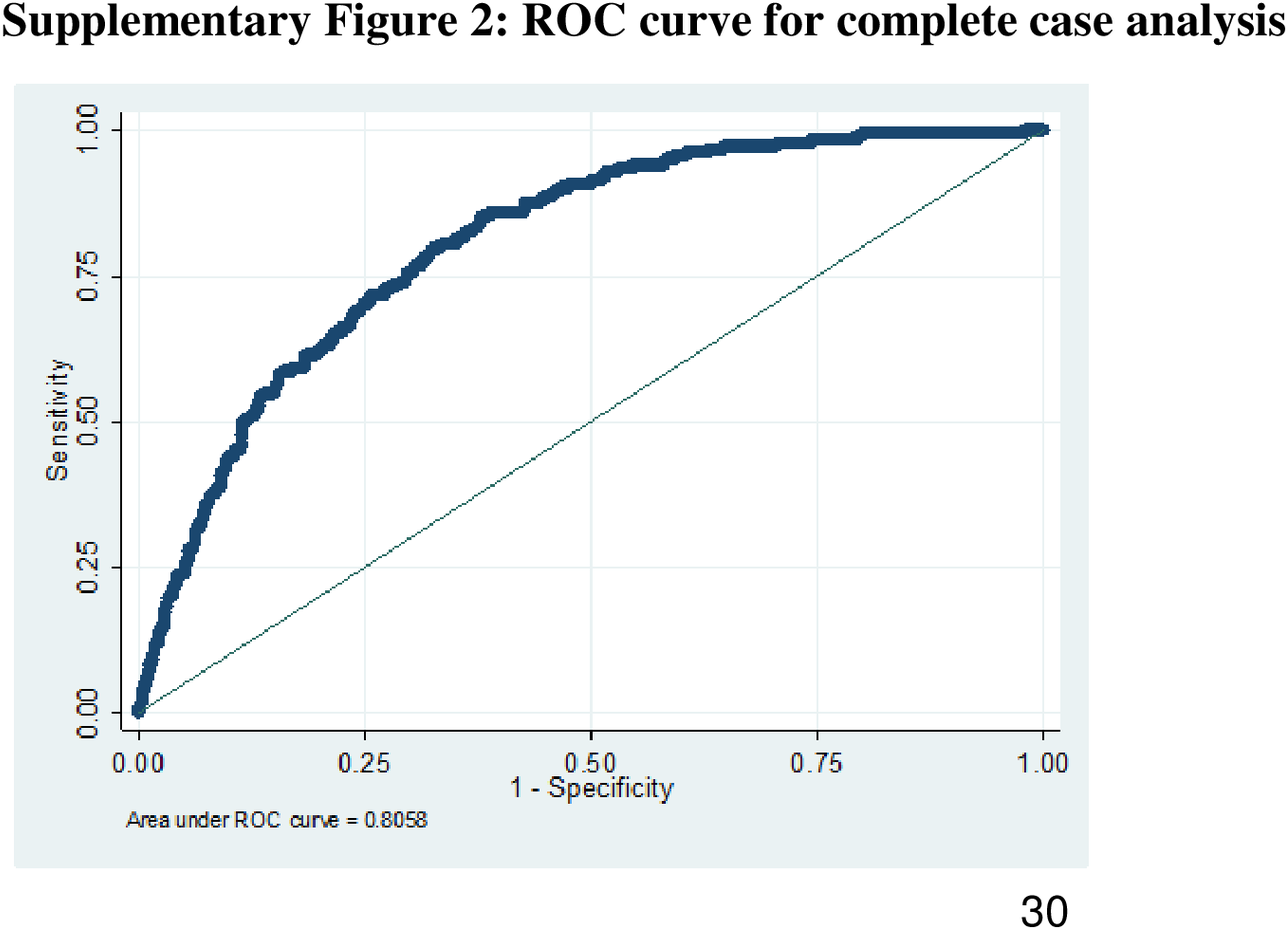

Supplement: S2 Fig — (TIF) [file pone.0244989.s002.tif]
