# Supplementary material for: Validation of a simplified risk prediction model using a cloud based critical care registry in a lower-middle income country
Source: PLoS One. 2020 Dec 31;15(12):e0244989. doi: 10.1371/journal.pone.0244989 (PMC7775074; doi:10.1371/journal.pone.0244989)
Supplement: S1 Table — (DOCX) [file pone.0244989.s003.docx]

**S1 Table. Variation of sensitivity and specificity at different cutpoints**

| Cutpoint | Sensitivity | Specificity | Correctly Classified |
| --- | --- | --- | --- |
| >=0.1 | 99.87% | 4.01% | 13.61% |
| >=0.2 | 88.60% | 60.78% | 63.57% |
| >=0.3 | 66.97% | 74.83% | 74.05% |
| >=0.4 | 59.97% | 81.11% | 78.99% |
| >=0.5 | 52.85% | 85.71% | 82.42% |
| >=0.6 | 48.70% | 90.14% | 85.99% |
| >=0.7 | 40.67% | 94.10% | 88.74% |
| >=0.8 | 23.19% | 96.78% | 89.41% |
| >=0.9 | 4.79% | 98.79% | 89.37% |
