# Supplementary material for: Validation of a simplified risk prediction model using a cloud based critical care registry in a lower-middle income country
Source: PLoS One. 2020 Dec 31;15(12):e0244989. doi: 10.1371/journal.pone.0244989 (PMC7775074; doi:10.1371/journal.pone.0244989)
Supplement: S2 Table — (DOCX) [file pone.0244989.s004.docx]

**S2 Table. Performance of the e-TropICS model ( complete case analysis)**

| **Performance item** | **Complete case analysis, n= 1780** |
| --- | --- |
| Probability, mean (SD) | 0.32(0.25) |
| Optimal cut-off probability | 0.37 |
| Sensitivity (at optimum cut-off) | 0.72 |
| Specificity (at optimum cut-off) | 0.74 |
| AUC (95% CI) | 0.81 (0.776-0.836) |
| H/L C-statistic (p) | 20.68(0.008) |
| Brier score (95% CI) | 0.15(0.14-0.16) |
