# Supplementary material for: Validation of a simplified risk prediction model using a cloud based critical care registry in a lower-middle income country
Source: PLoS One. 2020 Dec 31;15(12):e0244989. doi: 10.1371/journal.pone.0244989 (PMC7775074; doi:10.1371/journal.pone.0244989)
Supplement: S3 Table — (DOCX) [file pone.0244989.s005.docx]

**S3 Table. Number of patients included from each site:**

| No | Institution name | City | Number of participants |
| --- | --- | --- | --- |
| 1 | Apollo Speciality Hospital - OMR | Chennai | 567 |
| 2 | Apollo Main Hospital | Chennai | 454 |
| 3 | Apollo Cancer Institute | Chennai | 288 |
| 4 | IQRAA | Calicut | 236 |
| 5 | Apollo Speciality Vanagaram | Chennai | 314 |
| 6 | Apollo First Med Hospital | Chennai | 220 |
| 7 | Mehta Hospital | Chennai | 15 |
